# Thoughts, perceptions and concerns of coastal residents regarding the discharge of tritium-containing treated water from the Fukushima Daiichi Nuclear Power Plant into the Pacific Ocean

**DOI:** 10.1186/s12889-023-17349-1

**Published:** 2023-12-06

**Authors:** Varsha Hande, Makiko Orita, Hitomi Matsunaga, Yuya Kashiwazaki, Xu Xiao, Thierry Schneider, Jacques Lochard, Yasuyuki Taira, Noboru Takamura

**Affiliations:** 1 Department of Global Health, Medicine and Welfare, Atomic Bomb Disease Institute, Nagasaki University Graduate School of Biomedical Sciences, Nagasaki, 852-8523 Japan; 2 Nuclear Protection Evaluation Centre (CEPN), Fontenay-aux-Roses, France; 3 Department of Health Risk Control, Atomic Bomb Disease Institute, Nagasaki University, Nagasaki, Japan

**Keywords:** Fukushima, Tritium, Water, Radiation, Marine ecosystems, Public health

## Abstract

**Background:**

As a part of the decontamination process after the Fukushima Daiichi Nuclear Power Plant accident of 2011, 1.32 million tonnes of tritium-containing water will be discharged from the power plant into the Pacific Ocean. Although radiobiological impacts of the treated water discharge on the public and the environment were reported to be minimal, Tomioka and Okuma locals expressed unease regarding the long-term recovery of their towns, which are economically dependent on the agricultural, fishery, and tourism sectors. This study presents thoughts, perceptions and concerns of Tomioka and Okuma locals regarding the discharge of FDNPP-treated water containing tritium into the Pacific Ocean to facilitate a more inclusive decision-making process that respects local stakeholder interests.

**Methods:**

Conducted from November to December 2022, surveys were mailed to current residents and evacuees aged 20 years or older registered with the town councils.

**Results:**

Out of 1268 included responses, 71.5% were from those > 65 years. 65.6% were unemployed, 76.2% routinely visited hospitals, and 85.5% did not live with children. 61% did not want to return to Okuma/Tomioka. Anxiety about radiation-related health effects (38.7%), consuming food produced in Okuma/Tomioka (48.0%) and genetic effects (45.3%) were low. >50% reported poor physical and mental health. 40% were acceptive, 31.4% were unsure, and 29.7% objected to the discharge plans. Multinomial regression analysis revealed that, compared to acceptive responders, those who objected were more likely to be female, unemployed, and have anxiety about radiation-related genetic effects and poor mental health. Unsure responders were similarly more likely to be female, anxious about radiation-related genetic effects and have poor mental health.

**Conclusion:**

The poor mental health of the locals, connected to high levels of risk perception and anxiety about the loss of economic opportunities related to the discharge plans, must be addressed. The 30-year discharge process could handicap local industries and hamper post-disaster socioeconomic recovery due to the circulation of false rumours among consumers. These results highlight the need to actively involve residents in the towns’ recovery process to address local concerns. The focus should be on the judicious combination of transparent science with the human aspect of recovery and narratives highlighting dialogues between local stakeholders and experts to enable the locals and the general public to make informed decisions about their protection and future.

## Background

A critical obstacle in the recovery of Fukushima after the Great East Japan Earthquake and tsunami on March 11 2011, is the appropriate management of Advanced Liquid Processing System (ALPS)-treated water (henceforth termed “treated water”). Large volumes of seawater were sprayed into the Fukushima Daiichi Nuclear Power Plant (FDNPP) reactors to cool the fuel debris generated during the accident. After coming in contact with concentrated radioactive substances, the seawater became contaminated, the volume of the contaminated water has since been increasing after mixing with ground- and rainwater at the FDNPP site. To reduce the risk of radiation of FDNPP workers’ exposure to radiation in contaminated water, in August 2011, Tokyo Electric Power Company (TEPCO) started treating this water with filtration, desalination and in 2013, the ALPS system. Through these effects, all radioactive substances, barring tritium, were removed [1]. Since then, 1.32 million metric tonnes of this treated water has been stored in 1,066 storage tanks on site, but site capacity at the FDNPP has been rapidly diminishing. While TEPCO has taken various countermeasures against further volume increase, leakages and accidents in case of natural disasters, the need to remove the water from this site to advance the decommissioning and recovery process remains [2]. Accordingly, on April 13 2021, the Japanese government released a statement announcing the plan to discharge almost 1.32 million tonnes of ALPS-treated water into the Pacific Ocean over the next 30 years [3, 4].

As per the regulations set nationally by the Japanese government, internationally by the International Atomic Energy Agency, and the Nuclear Regulation Authority, and taking into account submitted public comments, radiobiological impact assessments of the treated water discharge on the public and environment were conducted. The results showed minimal effects [5–8]. Due to tritium’s short physical-half-life of 12.32 years, short biological half-life in the human body of 10 days, maximum energy of emitted beta-decay electrons of 18.6 keV and low production rates in nuclear reactors, the estimated exposure was calculated to be smaller than that of global fallout and with natural production [9]. The effective dose to the public who might frequent the sea around the discharge point was 0.00002–0.000002 mSv/year, and the impact on marine ecosystems present in the surrounding sea area was 0.000001–0.0000004 mGy/day [5]. The most recent maximum radioactivity concentrations of Cs-134/137 in fish sampled in February 2023 within a 20-km radius of the FDNPP was < 5.0 Bq/kg, and the most recent maximum radioactivity concentrations of Cs-134, Cs-137, H-3, and Sr-90 in seawater collected along the coast of the power plant were 0.93 Bq/cm3, 0.98 Bq/L, 0.55 Bq/L, and 0.0062 Bq/L, respectively [10].

The effective management of treated water is a crucial milestone in the mid-and long-term roadmap towards complete decommissioning of the FDNPP and recovery of Fukushima Prefecture. Although the plan for discharge was approved, neighbouring countries like China, South Korea, Taiwan and the Pacific Islands have expressed discontent with this decision and stated that they were not consulted during the decision-making process [11]. Various international environmental nongovernmental organisations and local Fukushima residents, particularly those involved in fisheries, voiced their doubts regarding the minimal long-term effects of prolonged exposure to a large amount of tritium on human health, the marine environment and global ecosystems [12, 13]. Local fisheries also expressed concerns regarding reputational damage and socioeconomic impacts caused by the discharge of water perceived as “contaminated” in commercial fishing zones [14, 15]. Those concerned accordingly proposed various discharge method alternatives, such as transport to a remote site or long-term storage of the treated water. The latter, however, required consent from the local landowners and local governments, who are also important stakeholders in this decision. Mayors of both Okuma and Futaba towns have resolutely opposed the proposal of long-term storage of treated water, with Okuma’s mayor stating, “There will be no change of use. It’s a betrayal of the townspeople who provided us with the land for reconstruction.” [16] It is thought that the stored water would act as a source of radioactive concern in the case of another natural disaster, hindering the return of past residents to their towns or the arrival of new ones.

Due to the protracted nature and significant social, psychological, and economic impacts of the discharge process, the inclusion and participation of local stakeholders in related decision-making processes are necessary to address concerns and safeguard regional interests. This is especially true for the residents of coastal towns along Fukushima, such as Tomioka and Okuma, who will face the daily and long-term effects of the treated water discharge. The evacuation order was lifted for 85% of the area in Tomioka in 2017, and for 40% of the area in Okuma in 2019, excluding the difficult-to-return zones, allowing residents to return to their hometowns. Since then, while Tomioka and Okuma have been endeavouring to achieve long-term socioeconomic recovery, locals feel that these discharge plans impede the recovery process by being a source of continuous risk [17]. Areas that exhibit long-term recovery from disasters are characterised by strong networks and active local governance [18], where the participation of residents has led to favourable changes in policy and outcomes [19, 20]. As coastal residents will be grappling with the daily consequences of the 30-year discharge process, examining their views, concerns and perceptions of potential risks is crucial [21, 22]. This research is part of the perspective of setting up a more integrated decision-making process that is more inclusive and respectful of the local interests of cities in the process of recovery. Presenting may contribute to a community dimension to the plan elaborated by the authorities.

## Methods

### Participants

This study was conducted in Okuma and Tomioka towns from November to December 2022. The target study participants were current residents and current evacuees still registered with the Okuma/Tomioka town council as of November 2022, aged 20 and over, and who can receive mailings from Okuma/Tomioka town council and have given their consent to participate in the study. The municipal office distributed two questionnaires to each household within the study area. We instructed that, if the household consisted of only one target participant, the second questionnaire be discarded. If there were three or more target participants in the household, additional questionnaires would be mailed.

Excluding residents aged less than 19 years, the total population of both towns combined was approximately 17,400 (8700 males and 8700 females), and the number of total households was approximately 10,400, according to national statistics. All study protocols were approved by the ethics committee of Nagasaki University Graduate School of Biomedical Sciences (approval No. 22,081,901, 5 September 2022).

### Questionnaire

The current questionnaire was based on the Fukushima Health Management Survey [23] and on previous studies conducted within these towns [24, 25]. It contained questions related to participant demographics (age, sex, current prefecture of residence, occupation, and if currently living with children below the age of 18), intention to return to Okuma/Tomioka, perception of radiation-related risks associated with living in Okuma/Tomioka (health effects, genetic effects, fear of consuming food produced from these towns), thoughts about the plan to discharge the treated water into the Pacific Ocean (acceptance, non-acceptance, unsure), specific worries regarding the discharge of treated water, desire to know specific information regarding the discharge of treated water, and preferred type of risk communication method. Responses were in the form of yes/no, or where relevant, as a multiple-choice answer. Responses were provided as 4-point scales (1 = strong yes, 2 = probably yes/a lot, 3 = probably no/a little, 4 = strong no). Risk perception was assessed using Lindell’s 4-point Likert scale [26, 27].

Quality of life was assessed using the validated Japanese version of the HR-QoL Short Form-8 (SF-8) scale [28, 29], which measures the health status of eight dimensions: general health, physical function, physical role (limitations in role due to physical health dysfunction), bodily pain, vitality, social function, mental health, and role emotional (limitations in role due to emotional health dysfunction). Answers were provided on a 5- or 6-point response scale, ranging from 1 (very good/not hindered at all) to 5 or 6 (very bad/inability to function). The SF-8 is interpreted based on scaled scores for two broad classifications: the Physical Component Summary (PCS; comprising general health, physical function, physical role, and bodily pain) and the Mental Component Summary (MCS; comprising vitality, social function, mental health, and role emotional). Scores higher than 50 ± 10 were considered good health, based on mean values among the general population in Japan [28].

### Statistical methods

The present study analysed the views of residents and evacuees on the decision to discharge treated water into the Pacific Ocean. Responder frequencies for each variable were first noted. The factors that played a significant role in responders belonging to each group (acceptance, non-acceptance, unsure) regarding the discharge of treated water were identified using the chi-square test. Pair-wise deletion of cases was done for factor analysis. Multinomial regression analysis was then used to determine the characteristics of responders based on their views of the decision to discharge treated water. Responders who chose not to disclose their sex (n = 2) were excluded from this analysis. Odds ratios (OR)s with 95% confidence intervals (95%CI) were obtained. Data analysis was performed using IBM SPSS Statistics version 28. p-values < 0.05 were considered statistically significant.

## Results

From 10,400 households, 1360 responses were received. After excluding responses due to missing data, a final sample of 1268 responders were examined regarding their thoughts on the discharge of treated water into the Pacific Ocean.

### Responder characteristics

Most responders were elderly (71.5%) and residing within Fukushima prefecture (74.8%). Males (50.3%) and females (49.5%) were equally represented among the responders. The majority of responders were unemployed (65.6%), but the most common form of employment was as a company employee (17.1%). Most responders routinely visited hospitals (76.2%) and did not currently live with a child (85.5%). Around 61% of responders did not want to return to Okuma or Tomioka, although the risk perception regarding radiation-related health effects (38.7%), consuming food produced from Okuma and Tomioka (48.0%) and radiation-related genetic effects (45.3%) were not high. Most responders reported poor physical (57.4%) and mental (54.3%) health. Although 84.6% of the sample indicated their feelings of reciprocity as high, the majority expressed no civic participation (67.4%) or social cohesion (33.3%) in their communities. (Table 1: Responder characteristics)

**Table 1:** Responder characteristics

Variable	Reference	N	%
Town (n = 1360)	Tomioka	691	50.8
	Okuma	669	49.2
Age (n = 1342)	< 60y	382	28.5
	≥ 60y	960	71.5
Sex (n = 1353)	Male	681	50.3
	Female	670	49.5
	Prefer not to say	2	0.1
Current residence (n = 1348)	Fukushima	1017	74.8
	Outside Fukushima	331	24.3
Employment status (n = 1340)	Unemployed	879	65.6
	Self-employed	53	4.0
	Company employee	229	17.1
	Civil servant	45	3.4
	Others	134	10.0
Regular hospital visits (n = 1351)	Yes	1029	76.2
	No	322	23.2
Living with children (n = 1337)	Yes	194	14.5
	No	1143	85.5
ITR (n = 1338)	Returned	119	8.9
	Yes	131	9.8
	Unsure	279	20.9
	No	809	60.5
Risk perception (health effects) (n = 1350)	Yes	522	38.7
	No	828	61.3
Risk perception (genetic effects) (n = 1341)	Yes	607	45.3
	No	734	54.7
Risk perception (food consumption) (n = 1351)	Yes	648	48.0
	No	703	52.2
Thoughts about discharge of treated water (n = 1349)	I accept it	525	38.9
	I do not accept it	400	29.7
	Unsure	424	31.4
Worried about discharge of treated water (n = 1350)	Yes	785	57.7
	No	565	41.5
^1^Worried specifically about the discharge of treated water and…	Human health impacts	562	41.3
	Marine ecosystem impacts	866	65.1
	Impact on occupation	60	4.4
	Safety inspection and marine monitoring methods	436	32.1
	Genetic impacts	501	36.8
Want to know more about treated water (n = 1328)	Yes	912	68.7
	No	416	31.3
^1^Want to know more specifically about…	Method of discharge into ocean	332	24.4
	Effects on human health	659	48.5
	Impact on marine ecosystems	845	62.1
	Safety of agricultural, forestry and fishery products	480	35.3
	Treatment methods other than ocean discharge	463	34.0
Preferred type of meeting with expert (n = 1256)	Individual	118	9.4
	Gatherings of 10	467	37.2
	Lectures > 10	671	53.4
PCS (n = 1314)	< 50	754	57.4
	≥ 50	560	42.6
MCS (n = 1314)	< 50	714	54.3
	≥ 50	600	45.7

^1^ Multiple response allowed

### Thoughts on the plan to discharge treated water into the Pacific Ocean

Around 40% of responders stated that they accepted the plans to discharge treated water, 31.4% said they were unsure, and the remaining 29.7% were unaccepting this decision. The majority of the responders who accepted the decision were male (*p* < 0.001), not living with children (*p* = 0.035), residing within Fukushima prefecture (*p* = 0.003) and currently unemployed (*p* < 0.001). This group of responders did not want to return to Okuma or Tomioka (*p* = 0.024) and had no or low perception of risk regarding radiation-related health effects (*p* < 0.001), genetic effects (*p* < 0.001) or consuming food produced in Okuma or Tomioka (*p* < 0.001). These responders were naturally not worried about the discharge of treated water (*p* < 0.001), but the majority, similar to the responders in the “unsure” or “unaccepting” group, had the desire to gain more information regarding the effects of this discharge on marine ecosystems (*p* < 0.001). More than half of the responders of the “accepting” group reported good mental health (*p* < 0.001), but 51.5% stated that their physical health was poor (*p* = 0.003). (Table 2: Factors related to thoughts on the plan to discharge treated water into the Pacific Ocean)

**Table 2:** Factors related to thoughts on the plan to discharge treated water into the Pacific Ocean

Variable	Reference	Acceptance*	Unacceptance*	Unsure	*P-value*
		n = 507	n = 374	n = 387	
Town	Tomioka (n = 685)	48.4	53.3	51.4	0.324
	Okuma (n = 664)	51.6	46.8	48.6	
Age	< 60y (n = 382)	31.3	24.7	29.2	0.093
	≥ 60y (n = 949)	68.7	75.3	70.8	
Sex	Male (n = 677)	63.7	44.3	39.8	< 0.001
	Female (n = 663)	36.3	55.4	60	
	Prefer not to say (n = 2)	0	0.3	0.2	
Current residence	Fukushima (n = 1007)	80.2	73.4	71.1	0.003
	Outside (n = 330)	19.8	26.6	28.9	
Occupation	Unemployed (n = 869)	59.7	71.6	66.7	< 0.001
	Employed (n = 460)	40.3	28.4	33.3	
Regular hospital visits	Yes (n = 1018)	75.6	77.2	75.3	0.801
	No (n = 322)	24.4	22.8	24.7	
Living with children	Yes (n = 194)	14.6	11.3	17.8	0.035
	No (n = 1132)	85.4	88.7	82.2	
ITR	Returned (n = 117)	11.6	7.1	6.9	0.024
	ITR+ (n = 130)	10.3	9.9	9.1	
	Unsure (n = 227)	16.9	23.7	23.2	
	ITR- (n = 804)	61.2	59.3	60.9	
Risk perception (health effects)	Yes (n = 521)	21.4	69.1	32	< 0.001
	No (n = 820)	78.6	30.9	68	
Risk perception (genetic effects)	Yes (n = 605)	25.7	73.1	43.8	< 0.001
	No (n = 728)	74.3	26.9	56.2	
Risk perception (food)	Yes (n = 646)	29.3	77.9	43.4	< 0.001
	No (n = 696)	70.7	22.1	56.6	
Worried about treated water	Yes (n = 780)	24.3	98.2	62.1	< 0.001
	No (n = 562)	75.7	1.8	37.9	
Want to know more about treated water?	Yes (n = 906)	57.8	87	64.9	< 0.001
	No (n = 412)	42.2	13	35.1	
Preferred type of expert meeting	Individual consultation (n = 116)	9.9	10.2	7.8	0.007
	Gathering of ≤ 10 people (n = 463)	33.3	43.8	35.5	
	Lecture with a large group (n = 468)	56.8	46	56.8	
PCS	< 50 (n = 747)	51.5	61.9	60	0.003
	≥ 50 (n = 559)	48.5	38.1	40	
MCS	< 50 (n = 711)	43.5	63	60.3	< 0.001
	≥ 50 (n = 595)	56.5	37	39.7	

* %, ^1^ multiple response allowed

### Multinomial regression analysis

A multinomial regression analysis was conducted, where the reference group was the “acceptance” responders regarding their thoughts on the plan to discharge treated water into the Pacific Ocean. Compared to this group, responders who were unaccepting of this decision had 1.693 times higher odds of being female (95% CI 1.253–2.289, *p* < 0.001), 1.507 times higher odds of being unemployed (95% CI 1.089–2.085, *p* = 0.013), 7.277 times higher odds of perceiving high risk regarding radiation-related genetic effects (95% CI 5.333–9.928, *p* < 0.001) and 1.751 times higher odds of reporting poor mental health (95% CI 1.291–2.373, *p* < 0.001).

Compared to the reference group, responders who were unsure about the decision had 2.324 times higher odds of being female (95% CI 1.757–3.074, *p* < 0.001), 1.919 times higher odds of perceiving high risk regarding radiation-related genetic effects (95% CI 1.433–2.570, *p* < 0.001) and 1.704 times higher odds of reporting poor mental health (95% CI 1.287–2.256, *p* < 0.001). (Table 3: Multinomial regression analysis)

**Table 3:** Multinomial regression analysis

Group^1^	Variable	Reference	Odds ratio	Lower 95% CI	Upper 95% CI	*p-value*
Unacceptance	Sex	Female	1.693	1.253	2.289	< 0.001
	Occupation	Unemployed	1.507	1.089	2.085	0.013
	Risk perception (genetic effects)	Yes	7.277	5.333	9.928	< 0.001
	PCS	< 50	1.125	0.823	1.537	0.460
	MCS	< 50	1.751	1.291	2.373	< 0.001
Unsure	Sex	Female	2.324	1.757	3.074	< 0.001
	Occupation	Unemployed	1.184	0.882	1.589	0.260
	Risk perception (genetic effects)	Yes	1.919	1.433	2.570	< 0.001
	PCS	< 50	1.172	0.879	1.563	0.279
	MCS	< 50	1.704	1.287	2.256	< 0.001

^1^ Reference group: Acceptance

## Discussion

While 38.9% of responders accepted the plans to discharge the treated water into the Pacific Ocean, 29.7% stated that they felt unsure, and 31.4% could not accept these plans. Compared to responders who thought the discharge plans were acceptable, those who were unaccepting had higher odds of being women (OR 1.693, 95% CI 1.253–2.289, *p* < 0.001), being unemployed (OR 1.507, 95% CI 1.089–2.085, *p* = 0.013), perceive a higher risk of radiation-related genetic effects (OR 7.277, 95% CI 5.333–9.928, *p* < 0.001) and report poor mental health (OR 1.751, 95% CI 1.291–2.373, *p* < 0.001). Similarly, those who were unsure had higher odds of being female (OR 2.324 times, 95% CI 1.757–3.074, *p* < 0.001), perceiving a higher risk of radiation-related genetic effects (OR 1.919, 95% CI 1.433–2.570, *p* < 0.001) and report poor mental health (OR 1.704 95% CI 1.287–2.256, *p* < 0.001).

Studies set in these affected towns reported similar results of females perceiving higher genetic risk and reporting poorer mental health scores, which were shaped by psychological distress from the disaster [30, 31]. Morioka et al. conducted interviews with radiation-protection activist networks in Tokyo, Fukushima, and Sendai, Japan, and found that risk perception was also higher among women outside Fukushima prefecture as well, owing to cultural values and the social context of gender values [32]. However, to our knowledge, no such study regarding the relationship between demographic features and the acceptance of discharge plans has yet been published.

Our results thus indicated that the responders who were unsure or unaccepting of the plans to discharge the treated water had higher likelihoods of perceiving radiation-related genetic effects, health effects, and fears of consuming food produced in these towns. When compared to an unaffected region, Kashiwazki et al. found that the perception of radiation-related genetic effects between Fukushima and Tokyo residents aged 20–59 in 2018 indicated that, while 54.6% of Fukushima residents perceived a high genetic risk, 61.3% from Tokyo perceived the same (χ2 = 3.867, df = 2, *p* < 0.05). One reason for the reduced risk perception in Fukushima could be the regular risk communication sessions with radiation experts that still occur in the affected prefectures [27]. However, Kashiwazaki et al.‘s sample represents a younger population compared to our responders, whose mean age was 77 years. It has been demonstrated that older people generally have a higher risk perception compared to the younger population [33, 34]. Therefore, even though the risk perception among Fukushima residents is lower, the concerns and uncertainties of the older population have been overrepresented in the present study. It is thought that those who were exposed to risk communication efforts perceive less risk and are less inclined to be opposed to, or at the very least, not feel unsure about, the discharge plans.

Responders who were unsure or unaccepting of the plans to discharge the treated water also reported relatively poorer mental health scores. Poor mental health was defined as scores reported below 50, based on standardised values in a national Japanese cohort [29]. For age-specific scores, the mean ± standard deviation was 51.50 ± 5.75 for Japanese individuals aged 70–79 years old [35]. A more recent study conducted in 2015 in the Chiba prefecture among 715 participants reported mean scores for those aged 65 and older as 51.4 ± 5.9 [36]. Although no statistically significant differences were demonstrated, both these means were slightly higher relative to our study mean of 48 ± 5.7. Our results also showed a difference between the scores of responders who accept the discharge and those who do not or who are unsure about the discharge. Therefore, general mental health has been demonstrated to be better in unaffected parts of Japan, as compared to Fukushima. The perception of radiation risk is closely tied to mental health and can be influenced by variables such as age, gender, past experiences, and psychological distress from the disaster. While these multiple factors shape individual risk perception and mental health [37–39] in a post-disaster setting, chronic PTSD and psychosocial disturbances were repeatedly proven to be associated with higher levels of risk perception [40, 41]. The interplay between poor mental health as a result of the long-term effects of the disaster can manifest as high perceptions of risk, intolerance of information uncertainty and paying selective attention to risk [31]. This high-risk perception of the discharge, and anticipation of fewer consumers and tourists in their towns due to harmful damage resulting from it could be factors contributing to the non-acceptance expressed by some responders. There is a need to acknowledge and follow up on the high levels of radiation-related risk perception and poor mental health in the local Tomioka and Okuma communities, particularly among women, to ensure that they do not become worse among this group. Promoting interactions with experts and authorities not only allows residents to gain knowledge and resolve uncertainties, it also provides opportunities for local stakeholders to bring attention to and formulate solutions that tackle reputational damage and are, more importantly, acceptable to locals.

After the FDNPP disaster and a protracted evacuation period, Tomioka and Okuma have been striving towards socioeconomic recovery. In the face of a lengthy decontamination and decommissioning period, these areas had to re-establish the collapsed socioeconomic infrastructures after the disaster to create sufficient economic opportunities and improve the quality of life for residents. However, with the announcement of the plans to discharge treated water into the Pacific Ocean, these coastal communities faced a further setback in their long-term rehabilitation. Regional industries expressed their dismay with the discharge plans due to rumours about potential harms and high levels of risk perception that might be held by their consumers [42], decreasing the number of visitors, reducing the demand for local products and yielding detrimental long-term socioeconomic impacts to this region [17]. While TEPCO [5], international authorities [15], and the national government [6] collectively confirmed the safety of the discharge process, the present study demonstrated that the uncertainty among the responders regarding the long-term effects of releasing large volumes of tritium-containing water over the coming decades, especially regarding its movements in the ocean and accumulation in the marine ecosystem, remained.

The most productive way of addressing local concerns tied to high levels of risk perception is establishing an open, two-way dialogue between experts and the responders. The confusion among residents in the aftermath of the 2011 disaster demonstrated the importance of experts in acknowledging local concerns regarding potential hazards and having conversations about worst-case scenarios [43]. Communicating risk-related information should be a bilateral process in which ethical concerns and concerns based on the local knowledge of affected stakeholders are considered. The aim of risk communication sessions is the promotion of a “practical radiological culture” [44], encouraging the provision of sufficient information to locals for making informed decisions rather than convincing them about scientific facts. Studies have revealed that while purely science-based discussions may hamper reaching resolutions [42], narrative evidence is helpful in the formulation of more widely accepted policies. Kobayashi et al. [45] revealed that risk communication among family, friends and acquittances, regardless of expert involvement, was more influential in post-disaster recovery among affected locals. While experts could provide factual knowledge, they must function more as advisors and facilitators rather than the main actors in risk communication sessions. To address this mistrust, forming an independent body composed of trusted local leaders who, besides acting as middlemen between the locals and experts, also partake in data collection and analysis is beneficial. The ETHOS project in Belarus following the Chornobyl nuclear accident was the first experience to demonstrate that the involvement of local actors in the management of their daily living conditions in an area contaminated by radioactivity not only improved the protection of the inhabitants against radiation as well as their quality of life but contributed positively to the collective efforts of the authorities [17]. The involvement of local representatives, non-conventional actors and humanities and social sciences experts was advantageous in the decision-making process for steering the discussions between experts and non-traditional agents. As recommended by the International Commission on Radiological Protection and the Nuclear Energy Agency, the involvement of local stakeholders in cooperative processes combining scientific expertise and knowledge of local living conditions is essential for the success of risk assessment and management in complex exposure situations of populations residing in contaminated areas [46–48].

All local stakeholders must be encouraged to participate in dialogue sessions to voice their opinions, partake in seafood sampling practices and engage in ocean monitoring exercises [49]. There is a resulting improved level of trust and confidence in results, improvement in public education, and fostering of social ties as residents face obstacles together. By allowing the affected residents to regain control over the decisions about their daily lives, their sense of dignity is preserved, which will improve their well-being and lead to policy changes favouring local interests [50–52]. Indeed, the expert committee for the discharge of treated water was composed dominantly of engineering and physical science experts and TEPCO. The fishery representatives complained that their involvement in the consultations occurred after the details of the discharge were finalised among other experts [12]. It will be economically beneficial for coastal towns to encourage collaborations between the government, the nuclear industry and fishermen who have local knowledge of the movement of fish and peak tourist season to decide on discharge plans collectively. It is vital to seek out and opportunistically promote the active participation of the residents in these discussions, especially those who hold relatively higher perceptions of risk and report poorer mental health (more likely to be women, as shown by our results) so that this group does not feel like their concerns are being ignored.

Limitations of our study included those common to cross-sectional surveys, such as undetermined causality, a low response rate, and response bias. While we attempted to improve the response rate by posting questionnaires to all eligible households within the two towns, a majority of this population has chosen to remain in their evacuation areas, due to better employment opportunities and infrastructure. As such, many are not inclined to participate in the recovery efforts of their former towns of residence. There is also a selection bias of elderly responders, due to the strong sentimental value they place on their hometown compared to the younger generation of residents [53]. In the present study, only 30% of the sample were aged less than 60 years, and only 35% were employed. Owing to this small sample size, firm conclusions regarding this group of responders cannot be derived. There were no significant differences in perceptions of the discharge plans between younger and older responders, but compared to those who were employed, those who were unemployed were significantly more likely to be unaccepting or unsure.

In a randomly generated telephone poll conducted by a national newspaper from a voter pool in August 2023, among 1,042 responses, 53% were in favour of the discharge plans and 41% were opposed [54]. In comparison to our sample, a higher national proportion agrees with the discharge plans (53% versus 39%).

It is thought that a relatively smaller proportion of the study sample agrees with the discharge plans, as these responders would be directly affected by the long-term social, psychological, and economic impacts of the discharge that hinder the socioeconomic recovery of the towns, rather than the perception of radiobiological risks. Indeed, the most pressing issue is the reputational damage that the fishery and tourism industries of these towns would face as a result of this discharge. Concerns have been raised, particularly by those employed in the fishery industry, due to the potential reputational damage to Fukushima seafood and its effects on their business [55]. With this in mind, it is thought that responders who did not participate in the survey would likely be less interested in the discharge plans or have no major objections. Our results generally represent the views of the elderly locals who were directly affected by the nuclear accident and the impending discharge plans.

## Conclusions

Our results revealed that, compared to responders who thought the discharge plans were acceptable, those who were unaccepting had higher odds of being women, being unemployed, perceiving a higher risk of radiation-related genetic effects and reporting poor mental health. Similarly, those unsure had higher odds of being female, perceiving a higher risk of radiation-related genetic effects and reporting poor mental health. The discharge of treated water is a long-term problem that will persist until the completion of the decontamination and decommissioning processes. The results of this study are valuable to better address residents’ concerns regarding the decision to discharge treated water into the surrounding ocean. This study also suggests a possible strategy for involving residents in the long-term recovery process. The current focus should be on the judicious combination of transparent science with the human aspect of recovery and the inclusion of narratives emphasising dialogues between local stakeholders and experts with the aim of enabling the locals and general public to make informed decisions about their protection and their future. Additional research on the conditions and means of implementing an effective dialogue between experts and local stakeholders should make it possible to influence policies in order to take better account of local interests. Given the interest and also the concerns expressed at the international level, such a dialogue should also be conducted with foreign stakeholders and experts.

## Data Availability

All data are available from the corresponding author on reasonable request.
